# Supplementary figures and images for: Variable effects of transient Wolbachia infections on alphaviruses in Aedes aegypti
Source: PLoS Negl Trop Dis. 2024 Nov 4;18(11):e0012633. doi: 10.1371/journal.pntd.0012633 (PMC11575829; doi:10.1371/journal.pntd.0012633)

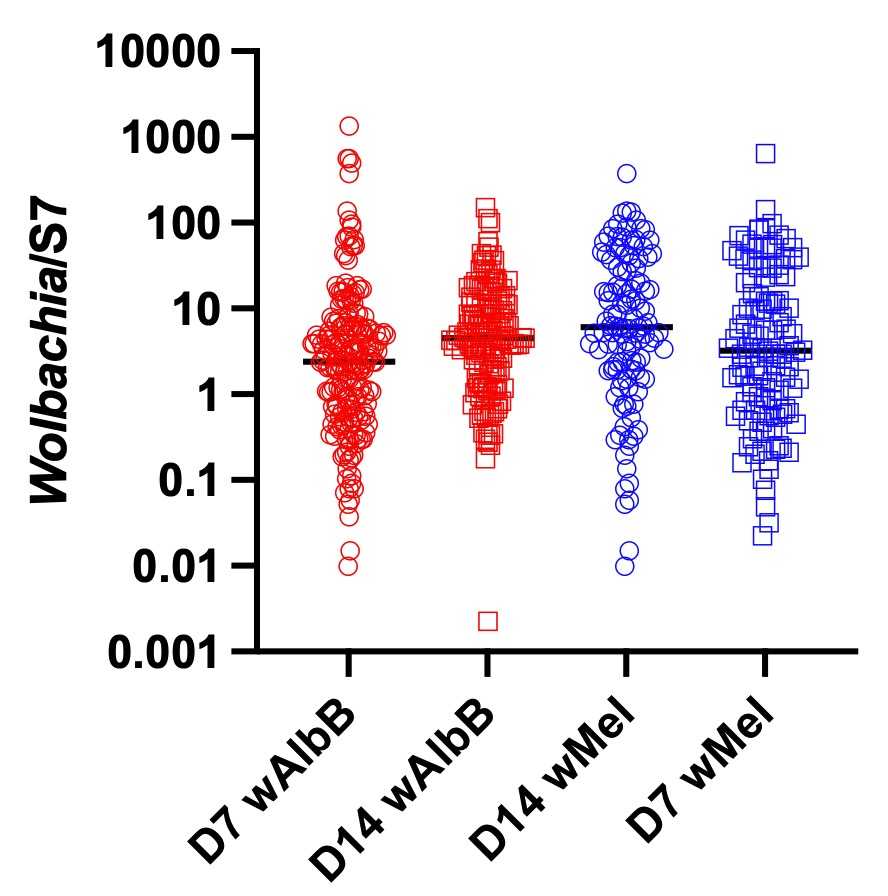

Supplement: S1 Fig — Groups are not statistically different (ANOVA, P = 0.39). (JPG) [file pntd.0012633.s001.jpg]
