# Supplementary material for: Variable effects of transient Wolbachia infections on alphaviruses in Aedes aegypti
Source: PLoS Negl Trop Dis. 2024 Nov 4;18(11):e0012633. doi: 10.1371/journal.pntd.0012633 (PMC11575829; doi:10.1371/journal.pntd.0012633)
Supplement: Attachment — Submitted filename: Response to Reviewersnew.docx [file pntd.0012633.s003.docx]

Below is the summary of key issues that need to be addressed in your revised manuscript version.

Explicitly emphasizing that the Wolbachia infections in your study were transient (in the title, abstract and introduction).

**Response: Done**

Providing the rationale for testing alphaviruses for which Aedes aegypti has not been implicated as an important vector.

**Response: These viruses were used not because Aegypti is a major vector of them in the field, but rather as a laboratory model to look at the effect of transient Wolbachia infections on a diversity of alphaviruses.**

Providing the sample sizes for each experimental group in the text and figures; discussing the power to detect the Wolbachia effects given the sample sizes.

**Response: Done**

Editor’s comment:

One experimental approach that comes to mind, which would be, in my opinion, more rigorous for testing the Wolbachia effects is the use of mosquito strains derived from the recently caught field mosquitoes infected with wMel or wAlbB and comparing them to their cured and transiently-infected counterparts. Could you discuss why such (or similar) experimental design was not employed?

**Response: We agree that testing infected mosquitoes from release sites would be ideal. However, the World Mosquito Program has threatened the lead author with punitive legal action if they collect mosquitoes from release sites. While these threats are ridiculous, legally baseless, unethical, and against current open scientific practices, they represent a level of additional hassle that we did not want to deal with at this point in time (none of us has time, energy, or money to defend against a lawsuit, no matter how baseless). Thus, transient infections were used.**

Comments from the reviewers:

Reviewer #1: Comments regarding the methods according to some of the questions above.

"Rockefeller mosquitoes were used to test ONNV and SINV, while Liverpool animals were used to test MAYV". Why not using the same aegypti strain? Although lab strain, any impact on the results? Limitation of the study as these are lab strains and their vector competence would not be the same if field strains were tested…

**Response: Our Rockefeller mosquito colony (ONNV, SINV) crashed during the experiments, so we switched to Liverpool (MAYV) We are not directly or statistically comparing results from Rockefeller to Liverpool.**

"Generation of virus stock": any interest or preference to use SINV and OONV derived from plasmid construction compared to MAYV obtained from virus stock. Impact on the results? Limitation of the study.

**Response: An infectious clone of MAYV did not exist at the time these experiments were conducted and thus were limited to using virus derived from a stock.**

Reviewer #2: The authors reported variable effects of Wolbachia depending on the strain and the alphavirus tested. The results ranged from wMel strongly blocking Mayaro virus infections and suppressing viral titers to no significant effects observed for O’nyong-nyong virus. Interestingly, there was also an enhancement of Sindbis virus infection in wAlbB- and wMel-infected mosquitoes. Overall, the experiments were well-controlled and appropriate for addressing the objectives of the study. Adequate statistical analyses were performed, although it would be helpful to include the sample size in the figures or mention it in the figure legends.

--------------------


Reviewer #1:

Specific comments

"Ae. aegypti vector competence for alphaviruses SINV and ONNV". Please add a figure in sup file of this control experiment.

**Response: The sample sizes and outcomes for this experiment were already given in the results. We did not record titers for this pilot, only infection, dissemination, and transmission rates.**

The results presented in the figures 2 and 3: VC results of the control strain (control experiment) is not in the same range of the controls presented in the figures except for MAYV. Reproducibility of the results.

**Response: Variability in technical and biological replicates is common in these types of experiments. This is why we perform replicates.**

A bias in the experimental set-up is to have used two different aegypti strains. It would have been interesting to test at least once a virus in both aegypti strains, to exclude confounding impact

**Response: See comments above.**

Reviewer #1:

"Another possibility is the nature of the Wolbachia infections: this work used transient Wolbachia infections rather than stable, maternally inherited Wolbachia infections". Fig S1 showed an overall Wolbachia density. However full bodies have been analyzed. It would have been interesting to test legs or heads or… to be sure that injected Wolbachia in the thorax indeed disseminated in the whole mosquito including abdomen/midgut. Indeed with oral blood feeding, viruses has to pass through the midgut barriers and competition between Wolbachia and virus at this step is of prime importance.

**Response: We have performed and published these types of analyses many times in the past (i.e. Hughes et al. 2011, 2012, 2014 and many others). We are injecting Wolbachia into the hemolymph where it establishes infection in many somatic tissues. For example, here is wAlbB distribution (labeled in orange) in a somatically infected mosquitoes (From Hughes et al. 2011). Wolbachia is primarily established in the fat body, neural tissue, and hemocytes.**

"We also only examined a single viral genotype for each virus and did not compare multiple mosquito genotypes". And more Ae/MAYV results are different from SINV/ONNV/Ae: the last two experiments were conducted with both virus derived from plasmids and rockfeller strain.

**Response: See comments regarding plasmid vs. virus and mosquito strains above.**

Reviewer #2:

However, it is important to note that the comparison and extrapolation of effects from transient Wolbachia infection to the permanent infection used in Aedes aegypti field strategies may have been oversimplified. Cautious approach is warranted when applying the study's findings in practical applications. This concern is supported by the fact that the importance of Aedes aegypti as a vector varies among the different alphaviruses tested. Hence, considering the context-specific nature of these interactions is essential for a comprehensive understanding.

**Response: We are presenting lab results in this study; we are not directly addressing practical applications. These data may be useful for those who are attempting practical applications but that is beyond our specific scope.**

Reviewer #2: I have several suggestions and comments that I believe the authors should address in their manuscript.

Firstly, it is crucial for the authors to explicitly emphasize that the Wolbachia infection in their study was transient. This distinction is significant because although some studies have reported similar effects between transient Wolbachia infection and permanent infection, it is essential to avoid generalizing the findings to permanent infection scenarios. I recommend that the authors clearly state the transient nature of the Wolbachia infection the manuscript title and mentioning it in abstract to provide readers with a comprehensive understanding of the study design.
By addressing these points, the authors can provide a more accurate and nuanced perspective on the implications of their research, ensuring that readers fully understand the context and limitations of their findings.

**Response: Done.**

Minor comments:

Include the word transient in the title

**Response: Done.**

Include the word transient in the abstract

**Response: Done.**

Include smaple size in the figures or in the figure legends

**Response: Done.**

Given that the authors observed a strong blocking effect of wMel on Mayaro virus (MAYV) infections, and MAYV is the only alphavirus known to potentially be transmitted by Aedes aegypti in the field, it is crucial for the authors to discuss the implications of their findings more cautiously. Unlike Sindbis virus (SINV) and O'nyong-nyong virus (ONNV), where Aedes aegypti does not play a significant role in transmission to humans, the blocking effect observed in the case of MAYV has direct relevance to arbovirus transmission.  The authors should discuss the limitations and caveats associated with extrapolating these findings to other alphaviruses and real-world scenarios.

**Response: These viruses were used not because Aegypti is a major vector of them in the field, but rather as a laboratory model to look at the effect of transient Wolbachia infections on a diversity of alphaviruses.**

Reviewer #1: This work describes the vector competence for SINV, ONNV and MAYV for Ae aegypti transiently infected or not with wMel and wAlbB. Although interesting this work raised me a major remark: Why did the authors wanted to test these viruses on Ae. aegypti. Ae. Aegypti is not the major vector of these viruses. Studies have been shown that Ae. Aegypti can transmit SINV and ONNV, but it is very few and rely on specific Virus/Vector interactions. Except, for MAYV, Ae. Aegypti has been described as a potential vector along with lots of other one. Thus I am interrogating myself about the public heath interest of such experiments. Could the authors clearly justify their purposes and hypotheses.

**Response: These viruses were used not because Aegypti is a major vector of them in the field, but rather as a laboratory model to look at the effect of transient Wolbachia infections on a diversity of alphaviruses.**

Specific comments:

Introduction:
“In some cases, Wolbachia-infected animals….”: Why in some cases, if not in all cases please precise or adjust your sentence. Animals should be replaced by mosquitoes.

**Response: Wolbachia invasions are not always successful, both natural invasions and human-implemented invasions. Our wording is correct as stated.**

“However, native population replacement with Wolbachia-infected mosquitoes is not always successful
[16, 26-30].” These refs do not refer to unsuccessful replacement.

**Response: Some do refer to unsuccessful introductions (i.e. ref 16 where the mosquito genotype affected invasion).**

"Because previous studies have focused primarily on flaviviruses while neglecting
alphaviruses, we studied the alphaviruses Sindbis virus (SINV), O’nyong-nyong virus
(ONNV), and Mayaro virus (MAYV"). Indeed, but for none of these viruses, Ae. aegypti is an important vector.

"Multiple mosquito genera can transmit SINV but Culex and Culiseta are considered the primary vectors [47, 51, 58-59]." What about aedes? Same for ONNV?

**Response: These viruses were used not because Aegypti is a major vector of them in the field, but rather as a laboratory model to look at the effect of transient Wolbachia infections on a diversity of alphaviruses.**

Saying that:" In sum, these alphaviruses are a burden on global human health yet remain poorly understood, including if and how they are affected by Wolbachia". It would have been more interesting to test the effect of Wolbachia (is possible) in their major vectors.

**Response: Since Aedes aegypti is the main species where Wolbachia control is being implemented, it seems prudent to assess whether Wolbachia would affect viruses that they couple potentially be exposed to, whether they are implicated as major vectors or not.**

Reviewer #2: In this manuscript, Dodson et al. investigated the effects of a transient infection of two Wolbachia strains (wAlbB and wMel) on alphavirus protection in Aedes aegypti mosquitoes. Overall, the experiments were well-controlled and appropriate for addressing the objectives of the study. Adequate statistical analyses were performed, although it would be helpful to include the sample size in the figures or mention it in the figure legends.

**Response: Done.**
