# Supplementary material for: Variable effects of transient Wolbachia infections on alphaviruses in Aedes aegypti
Source: PLoS Negl Trop Dis. 2024 Nov 4;18(11):e0012633. doi: 10.1371/journal.pntd.0012633 (PMC11575829; doi:10.1371/journal.pntd.0012633)
Supplement: Attachment — Submitted filename: ResponseDodson.docx [file pntd.0012633.s004.docx]

**Response**

I apologize for how long these final revisions took and feel an explanation is owed. When the manuscript was accepted earlier this year, I went to upload the raw data used for analysis and found several minor discrepancies between what was in the figures and what was in my data files. As I will not publish a paper that I am not 100% confident of, I needed to chase down the source of these discrepancies, across multiple co-authors on several continents during the summer when people were on leave and traveling.

The original figures for this paper were generated by a third-party graphic designer. After obtaining datasets from all relevant co-authors, and cross-checking them across one another for accuracy, it became obvious that the data file used by the graphic designer to generate the figures was slightly incomplete, leading to mismatch between some of the figures and the correct datafile. These errors did not affect any of the original results or the conclusions, bar some slight changes in P values.

As we are no longer contracted with the designer, I found it necessary to re-make the figures myself. I also eliminated some sub-figures and present the results in a table instead. These changes are purely stylistic and do not alter the manuscript.
